# Sulforaphane Inhibits Adhesion and Migration of Cisplatin- and Gemcitabine-Resistant Bladder Cancer Cells In Vitro

**DOI:** 10.3390/nu16050623

**Published:** 2024-02-23

**Authors:** Hui Xie, Jochen Rutz, Sebastian Maxeiner, Timothy Grein, Anita Thomas, Eva Juengel, Felix K.-H. Chun, Jindrich Cinatl, Axel Haferkamp, Igor Tsaur, Roman A. Blaheta

**Affiliations:** 1 Department of Urology and Pediatric Urology, University Medical Center Mainz, 55131 Mainz, Germany; xiehui0831@outlook.com (H.X.); rutzjochen@gmail.com (J.R.); anita.thomas@unimedizin-mainz.de (A.T.); eva.juengel@unimedizin-mainz.de (E.J.); axel.haferkamp@unimedizin-mainz.de (A.H.); igor.tsaur@med.uni-tuebingen.de (I.T.); 2 Department of Urology, Goethe-University, 60590 Frankfurt am Main, Germany; timothy.grein@kgu.de (T.G.); felix.chun@kgu.de (F.K.-H.C.); 3 Institute of Medical Virology, Goethe-University, 60596 Frankfurt am Main, Germany; cinatl@em.uni-frankfurt.de

**Keywords:** sulforaphane, bladder cancer, drug-resistance, chemotaxis, integrins, cadherins

## Abstract

Only 20% of patients with muscle-invasive bladder carcinoma respond to cisplatin-based chemotherapy. Since the natural phytochemical sulforaphane (SFN) exhibits antitumor properties, its influence on the adhesive and migratory properties of cisplatin- and gemcitabine-sensitive and cisplatin- and gemcitabine-resistant RT4, RT112, T24, and TCCSUP bladder cancer cells was evaluated. Mechanisms behind the SFN influence were explored by assessing levels of the integrin adhesion receptors β1 (total and activated) and β4 and their functional relevance. To evaluate cell differentiation processes, E- and N-cadherin, vimentin and cytokeratin (CK) 8/18 expression were examined. SFN down-regulated bladder cancer cell adhesion with cell line and resistance-specific differences. Different responses to SFN were reflected in integrin expression that depended on the cell line and presence of resistance. Chemotactic movement of RT112, T24, and TCCSUP (RT4 did not migrate) was markedly blocked by SFN in both chemo-sensitive and chemo-resistant cells. Integrin-blocking studies indicated β1 and β4 as chemotaxis regulators. N-cadherin was diminished by SFN, particularly in sensitive and resistant T24 and RT112 cells, whereas E-cadherin was increased in RT112 cells (not detectable in RT4 and TCCSup cells). Alterations in vimentin and CK8/18 were also apparent, though not the same in all cell lines. SFN exposure resulted in translocation of E-cadherin (RT112), N-cadherin (RT112, T24), and vimentin (T24). SFN down-regulated adhesion and migration in chemo-sensitive and chemo-resistant bladder cancer cells by acting on integrin β1 and β4 expression and inducing the mesenchymal–epithelial translocation of cadherins and vimentin. SFN does, therefore, possess potential to improve bladder cancer therapy.

## 1. Introduction

Bladder cancer is one of the most common solid cancers worldwide, with nearly 573,000 new cases occurring yearly [1]. The efficacy of tumor treatment is closely associated with the disease stage. Patients with non-muscle-invasive bladder cancer have a five-year overall survival of nearly 80% but are prone to a high rate of recurrence. Once the cancer has become muscle-invasive, the five-year survival rate drops to less than 40%, and once metastasized (stage T4), to less than 5% [2]. The treatment armamentarium has considerably grown in the last few years, including immune checkpoint inhibitors, targeted drugs and antibody–drug conjugates. However, cisplatin-based chemotherapy still remains the treatment of choice for bladder cancer or recurrent bladder cancer. Due to intrinsic or acquired resistance, therapy still fails in nearly 50% of patients. Furthermore, cisplatin application is accompanied by severe cardiotoxic, ototoxic, and nephrotoxic side effects.

Poor response and adverse effects drive many cancer patients to add natural plant extracts or ingredients to their treatment. The prevalence of herbal drug use in cancer patients is about 50%, with variations depending on socio-demographics and country. Reasons for consuming natural compounds are manifold and include symptom palliation, boosting the immune system, and/or improving conventional treatment [3,4]. Nevertheless, the high popularity of herbal “natural therapy” is not always supported by knowledge of the mode of action. Whether natural plant extracts or ingredients thereof do in fact improve or even worsen the response rate of bladder cancer patients to a cisplatin-based protocol has not been adequately evaluated.

The natural isothiocyanate sulforaphane (SFN) is found in its precursor form, glucoraphanin, in vegetables from the Brassicaceae family, with a high enrichment in broccoli sprouts. To activate SFN, the enzymatic hydrolysis of glucoraphanin by myrosinase is required. Preclinical and clinical studies have already documented antioxidant, immune-modulatory, and anti-apoptotic properties of SFN. Furthermore, SFN has been proven to be a chemoprotective agent by interfering in multiple signaling pathways related to tumor initiation, growth, and progression [5]. Due to its lipophilic nature and high bioavailability, SFN rapidly reaches peak plasma levels after 4 h [6]. These characteristics make SFN a promising candidate for clinical application with anti-cancer properties synergic with cisplatin having been reported. The present investigation explores the influence of SFN on the adhesion and migration behavior of bladder cancer cell lines with established cisplatin and gemcitabine resistance.

## 2. Materials and Methods

### 2.1. Cell Culture and Resistance Induction

The drug-sensitive cell lines RT4, RT112, and T24 were ordered from ATCC/LGC Promochem GmbH (Wesel, Germany), and TCCSUP was purchased from German Collection of Microorganisms and Cell Cultures GmbH (DSMZ; Braunschweig, Germany). The drug-sensitive tumor cells, termed “parental”, were all grown in cell culture medium, based on Isocove’s Modified Dulbecco’s Medium (IMDM; Gibco/Invitrogen, Karlsruhe, Germany), which was additionally supplemented with 10% fetal calf serum (FCS), 2% glutamax and 1% penicillin/streptomycin (all from Gibco/Invitrogen) in a humidified, 5% CO_2_ incubator. Cisplatin and gemcitabine resistance of RT4 (RT4^cis^, RT4^gem^), TCCSUP (TCCSUP^cis^, TCCSUP^gem^), T24 (T24^cis^, T24^gem^), and RT112 (RT112^cis^, RT112^gem^) were induced by exposing parental cells that were sensitive to cisplatin and gemcitabine (RT4^par^, TCCSUP^par^, T24^par^, RT112^par^) to increasing concentrations of cisplatin (Hexal, Holzkirchen, Germany) up to 1 µg/mL (TCCSUP, T24, RT112) or 2 µg/mL for RT4, or to increasing concentrations of gemcitabine (Hexal, Holzkirchen, Germany) up to 10 ng/mL (TCCSUP) or 20 ng/mL (RT4, T24, RT112). Resistance was verified by dose–response analysis [7].

### 2.2. Sulforaphane (SFN)

Both drug-sensitive and drug-resistant cell cultures were exposed to SFN at 20 µM. SFN was obtained from Biomol (Hamburg, Germany) as L-Sulforaphane. Control cell cultures did not receive SFN. To exclude the toxic effects of SFN, viability of the cell cultures was controlled by the trypan blue staining assay (Gibco/Invitrogen).

### 2.3. Integrin Expression

To analyze integrin surface expression, RT4, RT112, T24 or TCCSUP cells were detached from the culture flasks by enzymatic treatment with accutase (PAA Laboratories GmbH, Pasching, Austria). Subsequently, they were washed with 0.5% bovine serum albumin (BSA) (diluted in PBS; Gibco/Invitrogen), followed by incubation for 1 h at 4 °C with 20 µL of phycoerythrin (PE)-conjugated monoclonal antibodies, which included anti-integrin β1 (IgG1; clone MAR4) and anti-integrin β4 (IgG2a; clone 439–9B (all obtained from BD Biosciences, Heidelberg, Germany). Anti-phospho-integrin β1 (Thr788/789; Merck KGaA, Darmstadt, Germany) was labeled with allophycocyanin (APC). The integrin surface level was evaluated using a FACSCalibur flow cytometer (BD Biosciences; FL2-H (log) or FL4-H (log) channel histogram analysis; CellQuest Pro 4.0.2. software), whereby 1 × 10^4^ cells were analyzed per scan. Integrin expression was depicted as the mean number of fluorescence units. Unspecific fluorescence was evaluated by staining the cells with mouse IgG1-PE (MOPC-21), mouse IgG2a-PE (G155-178) or rat IgG2b-PE (R35-38; all from BD Biosciences).

### 2.4. Cell Attachment to Collagen and Fibronectin

Bladder cancer cell binding was assessed using 6-well multi-plates, either coated with collagen G type I (400 µg/mL dilution; Sigma-Aldrich, Taufkirchen, Germany), or with human plasma fibronectin (50 µg/mL dilution; BD Biosciences). Uncoated plastic dishes were used to detect unspecific cell binding and served as the controls. The multi-plates were washed twice with BSA (1%). Then, bladder cancer cells (treated vs. non-treated, resistant vs. sensitive) were added at a concentration of 0.5 × 10^6^ cells/well for 60 min at 37 °C. Subsequently, the plates were again washed to remove those cells which did not adhere. Adherent cells establishing firm contact to the well bottom were then fixed with glutaraldehyde (1%; Sigma-Aldrich) and counted microscopically (×200 magnification) in five different fields (0.25 mm^2^) using a raster ocular. The mean number of adherent cells in the five fields was determined.

### 2.5. Chemotaxis

The chemotaxis assay was based on the Boyden chamber method with an established serum gradient. TCCSUP, T24, or RT112 cells (0.5 × 10^6^ cells/mL), pre-treated with SFN, were added to the upper chamber of the transwell system (Greiner Bio-One, Frickenhausen, Germany), which was separated from the lower chamber by a membrane with a pore size of 8 µm. The upper chamber was filled with serum-free medium. RT4 cells did not migrate and were, therefore, not included in these experiments. The lower chamber contained medium enriched with 10% fetal bovine serum (FBS) as the chemoattractant. After overnight incubation, cells which did not migrate underneath the membrane were removed from the upper membrane surface with a cotton swab. Cells which migrated through the membrane and attached to the lower membrane surface were stained with hematoxylin and counted microscopically (×200 magnification). The mean number of migrated cells was determined from counts in five different observation fields.

### 2.6. Western Blot

Western blot analysis concentrated on proteins involved in tumor cell differentiation and migration processes and included E- and N-cadherin, vimentin and cytokeratin (CK) 8/18 expression. Tumor cell lysates were applied to a polyacrylamide gel and run for 90 min at 100 V. The concentration of acrylamide ranged from 3% to 12%, depending on the evaluated protein. The proteins were then transferred to nitrocellulose membranes for 1 h at 100 V and blocked with non-fat dry milk (1 h). Overnight incubation was finally carried out with monoclonal antibodies directed against E-cadherin, N-cadherin, vimentin, and CK 8/18 (all were from Cell Signaling, Leiden, The Netherlands). As secondary antibodies, HRP-conjugated goat anti-mouse IgG and HRP-conjugated goat anti-rabbit IgG were used at a 1:5000 dilution (both were from Upstate Biotechnology, Lake Placid, NY, USA). Proteins were visualized by the ECL detection reagent (Amersham/GE Healthcare, München, Germany) and analyzed with the Fusion FX7 system (Peqlab, Erlangen, Germany). Internal controls were carried out with an anti-β-actin antibody (clone AC-15; Sigma-Aldrich). To quantify the intensity of the protein bands, the protein/β-actin intensity ratio was quantified using GIMP 2.8 software.

### 2.7. Cadherin and Vimentin Localization

Cellular E-cadherin (RT112), N-cadherin (RT112, T24) and vimentin (T24) were localized by fluorescence microscopy. RT112 and T24 bladder cancer cells were treated with SFN, enzymatically detached from the culture flasks, and transferred to Falcon^®^ culture slides (8-well glass slides with polystyrene vessels; Merck Millipore, Darmstadt, Germany). Following attachment, cell specimens were washed twice and fixed in an ice-cold (−20 °C) methanol/acetone mixture (60/40 *v*/*v*). For visualization, cells were incubated with Alexa Fluor 488-conjugated E-cadherin, N-cadherin or vimentin monoclonal antibodies for 60 min (Cell Signaling). To preserve fluorescence, the cells were embedded in Vectashield antifade mounting medium (Biozol, Eching, Germany) and viewed under a fluorescence microscope (×630 magnification; Zeiss, Jena, Germany).

### 2.8. Blocking

RT112, T24, or TCCSUP cells were incubated with a function-blocking anti-integrin β1 (clone 6SG; Merck Millipore) or anti-integrin β4 antibody (clone ASC-8; both Merck Millipore), each for 60 min at a concentration of 10 μg/mL. Controls were not blocked. Subsequently, chemotaxis was carried out as described above.

### 2.9. Statistics

All experiments were carried out three to six times. Statistical significance was calculated with a *t*-test or ANOVA. Differences were considered statistically significant at a *p*-value of <0.05.

## 3. Results

### 3.1. Integrin Expression

**Figure 1:**
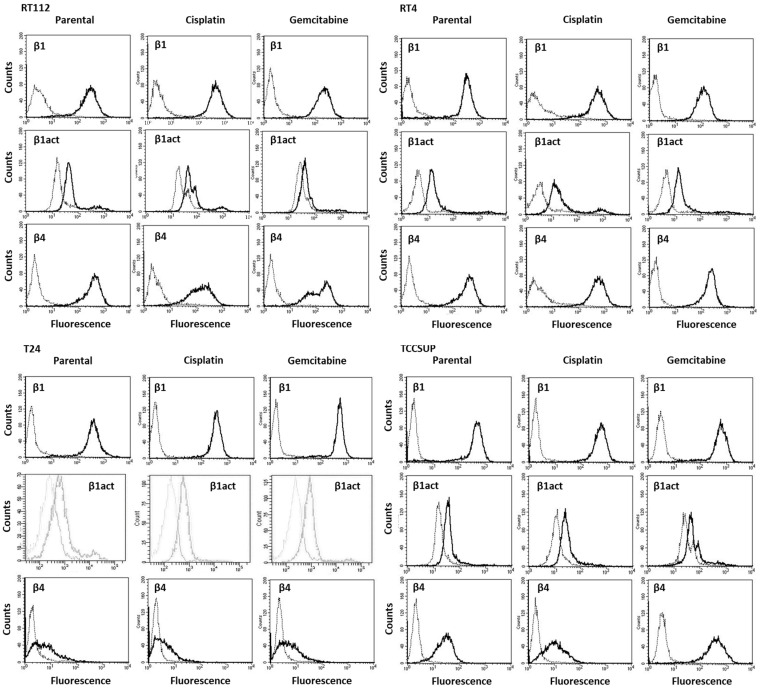
Integrin subtype expression on parental (sensitive to chemotherapy) and cisplatin- or gemcitabine-resistant RT112, RT4, T24, and TCCSUP cells. Single representative of three separate experiments. Solid line shows specific fluorescence; dashed line shows isotype IgG1.

Integrin β1, activated integrin β1, and integrin β4 were all expressed in the chemo-sensitive parental (^par^)and chemo-resistant (^res^) tumor cell lines with cell-specific differences (Figure 1). Little expression of integrin β4 on T24 cells was noted compared to TCCSUP, RT4, and RT112. The β4 level on TCCSUP^gem^ exceeded that of TCCSUP^par^ and TCCSUP^cis^ cells, and the β4 level on RT4^cis^ was higher than the level for RT4^par^ and RT4^gem^.

**Figure 2:**
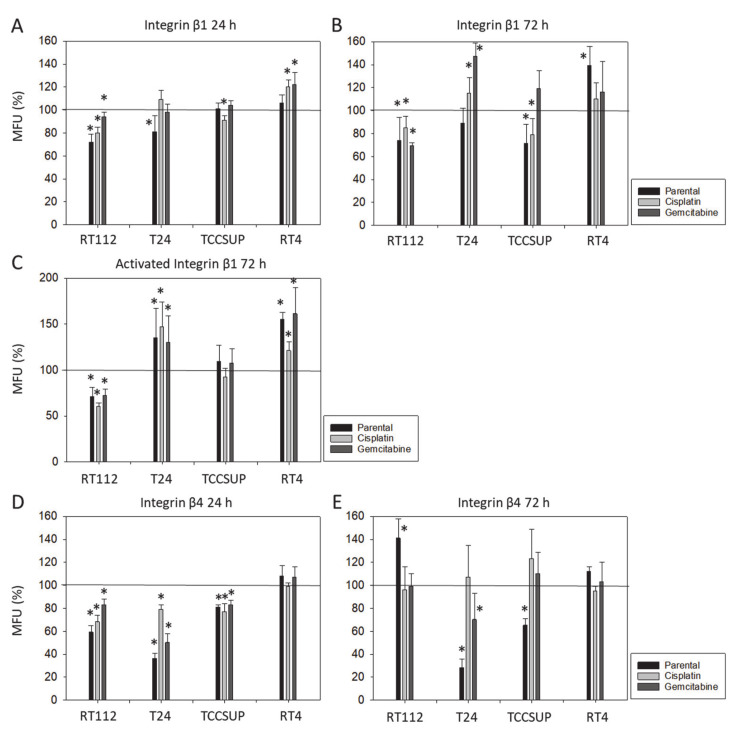
Integrin β1 (total and activated) and β4 expression on parental (sensitive to chemotherapy), cisplatin-resistant, gemcitabine-resistant RT112, T24, TCCSUP, and RT4 cells following SFN exposure (20 µM) for 24 and 72 h ((**A**): total β1 24 h, (**B**): total β1 72 h, (**C**): activated β1 72 h, (**D**): total β4 24 h, (**E**): total β4 72 h). Values are means relative to controls not treated with SFN (100%, marked by a horizontal line). MFUs = mean fluorescence units. Error bars indicate SD. * = significant difference to controls not treated with SFN; *n* = 3.

Treatment with SFN for 24 or 72 h altered integrin expression levels differently (Figure 2). Changes in integrin β1 were more pronounced after 72 h than after 24 h, with a down-regulation on RT112 (all sublines), TCCSUP^par^, and TCCSUP^cis^ cells, and an up-regulation on RT4^par^, T24^cis^ and T24^gem^ cells. Accordingly, activated integrin β1 decreased on RT112 (all sublines) and increased on RT4 (all sublines). An increase was also recorded on T24 (all sublines), whereas no significant changes were seen for TCCSUP (all sublines). In contrast to integrin β1, modifications of integrin β4 were stronger after 24 h than after 72 h. Integrin β4 was suppressed on RT112 (all sublines), T24 (all sublines), and TCCSUP (all sublines), but not altered on RT4.

### 3.2. Tumor Cell Adhesion and Chemotaxis

**Figure 3:**
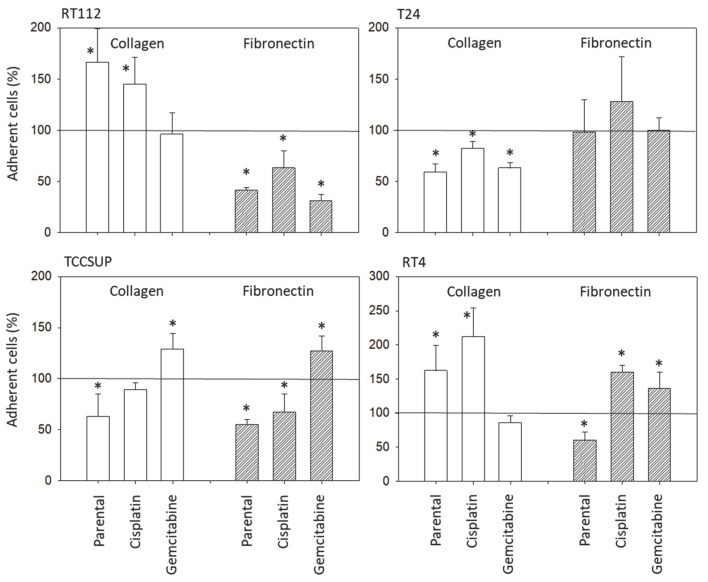
Influence of SFN (20 µM) on adhesion of parental (sensitive to chemotherapy), cisplatin-resistant, gemcitabine-resistant RT112, T24, TCCSUP, and RT4 cells to a collagen or fibronectin matrix. Counts from five fields of 0.25 mm^2^ (means ± SD, *n* = 4). All values are related to cells treated with SFN and expressed as a percentage thereof. Untreated cells served as controls and were set to 100% (marked by horizontal line). * indicates significant difference to controls not treated with SFN.

SFN significantly altered tumor cell adhesion. Adherence of RT112^par^ and RT112^cis^, as well as of RT4^par^ and RT4^cis^ to collagen, increased in the presence of SFN but was diminished for T24 (all sublines), all in comparison to untreated controls (Figure 3). Interestingly, less TCCSUP^par^ but more TCCSUP^gem^ was bound to collagen when exposed to SFN. Similarly, the binding of TCCSUP^par^ as well as of TCCSUP^cis^ to immobilized fibronectin was diminished, whereas the binding of TCCSUP^gem^ was enhanced. SFN inhibited attachment to fibronectin in RT112 (all sublines), but enhanced RT112 binding to collagen. The binding of RT4^par^ to fibronectin was reduced by SFN but elevated in the resistant sublines. No significant effects of SFN were observed regarding T24 attachment to fibronectin.

**Figure 4:**
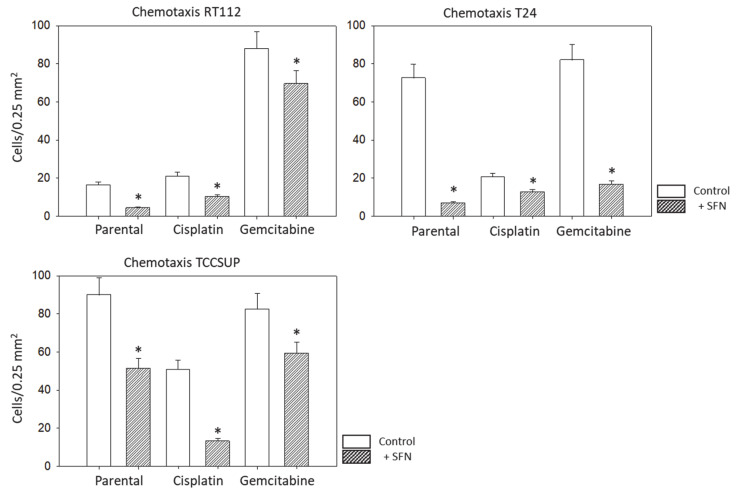
Influence of SFN (20 µM) on chemotaxis of parental (sensitive to chemotherapy), cisplatin-resistant, gemcitabine-resistant RT112, T24 and TCCSUP cells towards an FBS gradient. Counts from 5 separate 0.25 mm^2^ fields (means ± SD, *n* = 4); * indicates significant difference to controls not treated with SFN.

Chemotaxis was evaluated only for RT112, T24, and TCCSUP cells since RT4 cells did not migrate. SFN significantly lowered the migration of all three cell lines, whether they were sensitive or resistant to chemotherapy (Figure 4). The trypan blue dye exclusion test did not reveal signs of toxicity, which could have altered binding or migration behavior of the tumor cells.

### 3.3. Tumor Cell Differentiation

**Figure 5:**
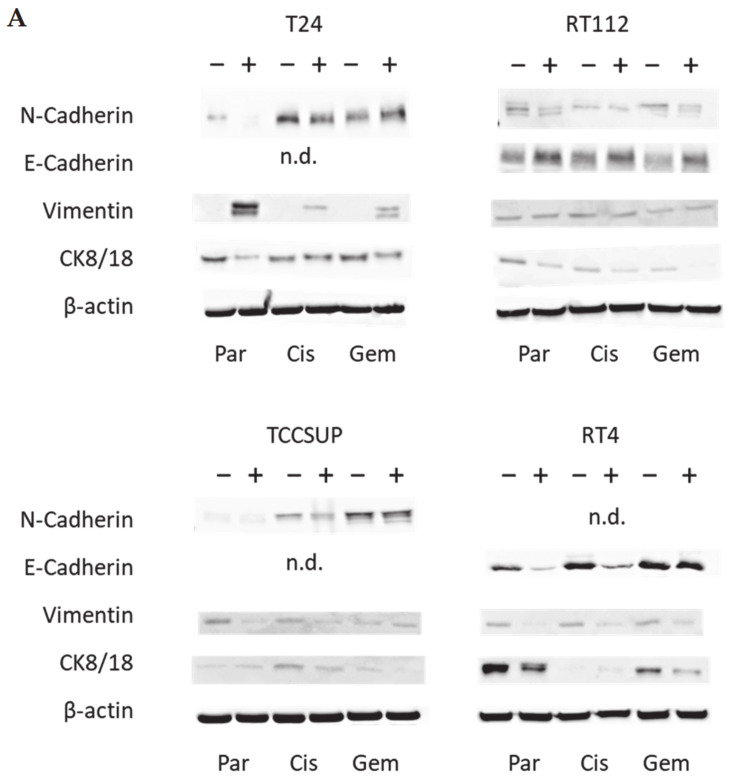

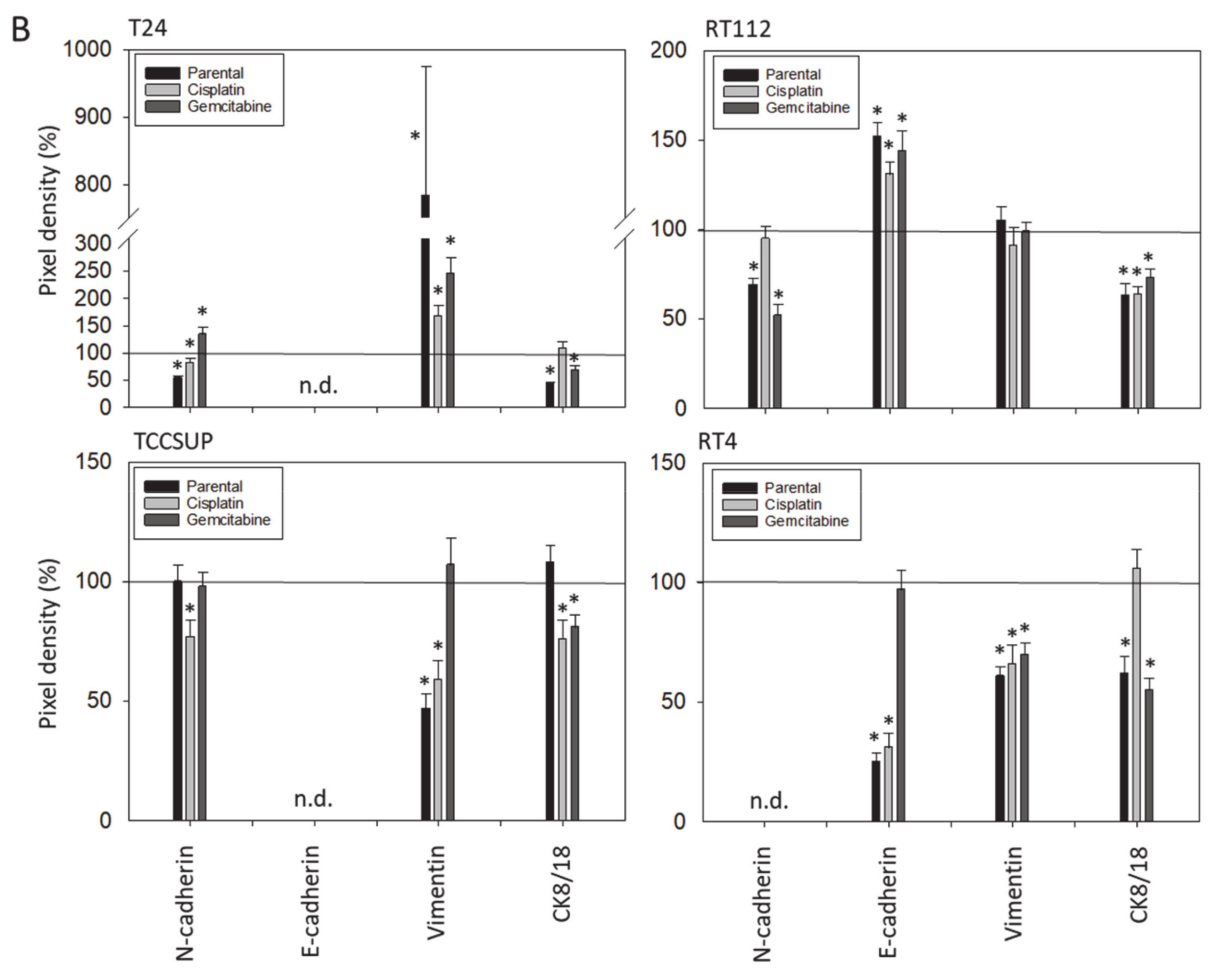
(**A**) Western blots of differentiation-related proteins in parental (sensitive to chemotherapy), cisplatin-resistant, gemcitabine-resistant RT112, T24, TCCSUP, and RT4 cells following SFN (20 µM) exposure for 24 h. All bands are representative of *n* = 3. β-actin was used to control protein loading and is representatively shown once. In total, 50 µg was used per sample. − indicates untreated controls, + indicates SFN treated cells. n.d. = non-detectable. (**B**) pixel density. All values are expressed as a percentage, related to control cells (set to 100% and indicated by a horizontal line), not treated with SFN. * indicates significant difference to controls.

Since E-cadherin, N-cadherin, vimentin, and CK 8/18 all contribute to cell adhesion, migration, and differentiation, their expression under the influence of SFN was examined (Figure 5 and Figure S1). The N-cadherin protein level was diminished in T24^par^, T24^cis^, RT112^par^, RT112^gem^, and TCCSUP^cis^ cells. Only a slight expression was apparent in TCCSUP^par^ cells, and N-cadherin was not detectable in RT4 cells. E-cadherin was not detectable in T24 (all sublines) or TCCSUP cells (all sublines). However, up-regulation in the presence of SFN was evident in RT112 cells (all sublines). E-cadherin was diminished in RT4^par^ and RT4^cis^ cells. Vimentin was also differently influenced by SFN in the different cell lines. SFN induced an elevation in T24 cells (all sublines), most prominently in T24^par^ cells. No differences were seen between SFN-treated and untreated RT112 cells. However, vimentin expression was lowered by SFN in RT4 (all sublines), TCCSUP^par^, and TCCSUP^cis^ cells. CK8/18 was suppressed by SFN in T24^par^, T24^gem^, RT112 (all sublines), TCCSUP^cis^, TCCSUP^gem^, RT4^par^, and RT4^gem^, with the overall expression level of CK8/18 being low in RT112 and TCCSUP cells.

### 3.4. Cadherin and Vimentin Translocation

**Figure 6:**
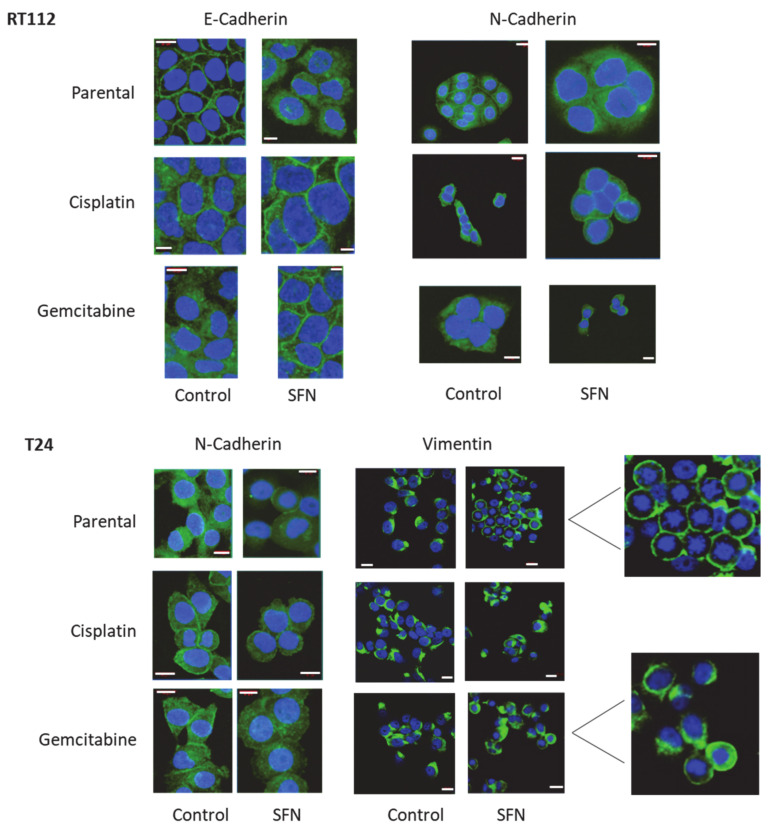
Distribution of E-cadherin and N-cadherin in parental (sensitive to therapy), cisplatin-resistant, gemcitabine-resistant RT112 cells and of N-cadherin and vimentin in T24 cells after 24 h SFN exposure (20 µM). Pictures taken by fluorescence microscopy (×630 magnification, oil immersion objective). White scale bar = 20 µm. Blue shows cell nuclei, green shows cadherin or vimentin staining.

Since E- and N-cadherin were detected in RT112 cells, localization was determined. In T24 cells, vimentin was evaluated, since strong SFN-induced alterations were apparent in Western blots. E-cadherin was exclusively localized at the cell surface membrane in RT112^par^ cells but was translocated into the cytoplasm when treated with SFN. In contrast, E-cadherin was enriched in the cytoplasm of RT112^cis^ and RT112^gem^ cells, but translocated to the cell membrane under SFN. Similar behavior was seen with the N-cadherin distribution in SFN-exposed vs. unexposed RT112 cells. However, the effects were not as prominent as those observed with E-cadherin (Figure 6). In contrast, N-cadherin was predominately expressed in the cytoplasm of T24^par^ and on the cell surface membrane of T24^cis^ and T24^gem^ cells under control conditions, but was then translocated to the cell membrane (T24^par^) or into the cytoplasm (T24^cis^, T24^gem^) after SFN exposure. Vimentin was enriched in one cytoplasmic section of T24^par^ cells and then evenly relocated to the membrane following treatment with SFN. This action was not observed in T24^cis^ and T24^gem^ cells.

### 3.5. Integrin Blocking Studies

**Figure 7:**
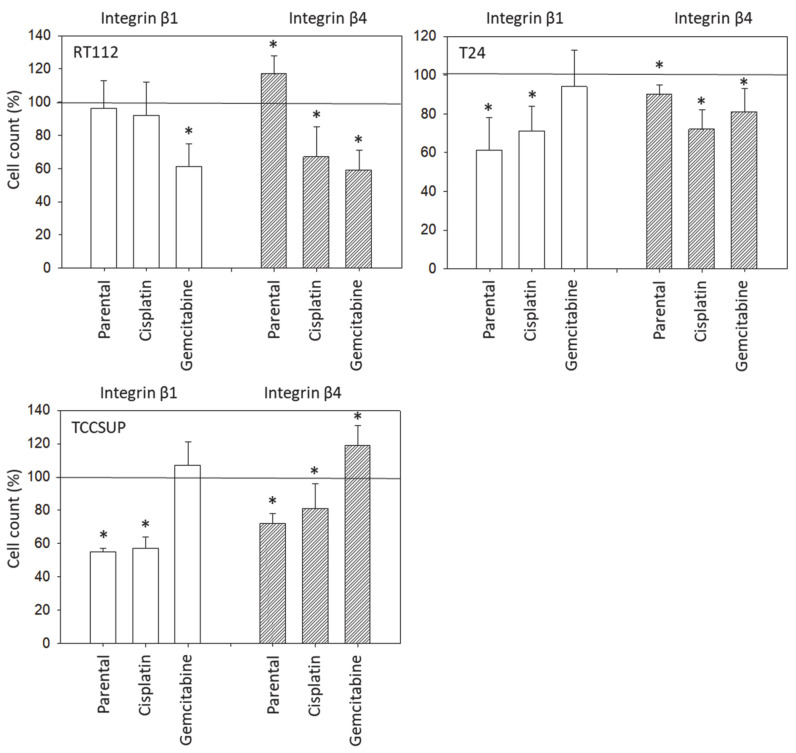
Influence of integrin β1 and β4 on the chemotactic movement of parental (sensitive to chemotherapy), cisplatin-resistant, gemcitabine-resistant RT112, T24, and TCCSUP cells towards an FBS gradient. Cell number expressed relative to unblocked controls (100%). Error bars indicate standard deviation; * = *p* ≤ 0.05; *n* = 3.

Blocking integrin β1 was associated with reduced chemotaxis of RT112^gem^, but not of RT112^par^ and RT112^cis^ cells (Figure 7). In contrast, blocking integrin β1 on T24 and TCCSUP suppressed the chemotaxis of parental and cisplatin-resistant sublines but did not suppress chemotaxis in T24^gem^ and TCCSUP^gem^ cells. Blockade of integrin β4 diminished chemotaxis of all cisplatin-resistant sublines. Chemotaxis of RT112^gem^ and T24^gem^ was also decreased, but that of TCCSUP^gem^ was elevated compared to the controls. Blocking integrin β4 down-regulated T24^par^ and TCCSUP^par^, whereas RT112^par^ cells were up-regulated.

## 4. Discussion

SFN (20 µM) significantly inhibited chemotaxis in sensitive, cisplatin- and gemcitabine-resistant bladder cancer cell lines in vitro. This concentration has been shown to inhibit the chemotaxis and migration (scratch assay) of sensitive T24 cells [8]. However, lower concentrations of 2.5 µM or 5 µM SFN may also be effective in suppressing bladder cancer cell motility in chemo-sensitive cells [9], although not as strongly as higher concentrations [10]. SFN has also been shown to diminish the migratory potential of cancers other than bladder cancer such as lung (10–40 µM SFN) [11], colorectal (10 µM SFN) [12], and breast (10–40 µM SFN) cancer [13]. Although these studies were not carried out on chemo-resistant tumor cells, they do show that SFN blocks migration in different cancers in vitro.

Aside from the potential relevance of SFN in treating chemo-sensitive cancer, the present investigation shows that SFN also inhibits chemo-resistant bladder cancer cells. The migration of all investigated cell lines, whether cisplatin- or gemcitabine-resistant or not, was significantly reduced after SFN exposure. The growth and proliferation of a panel of cisplatin- and gemcitabine-resistant bladder cancer cell lines have already been shown to be inhibited by SFN [7], indicating that it has inhibitory effects on other aspects contributing to tumor progression. Since acquired resistance and metastasis are major obstacles to successful cancer treatment, SFN could enhance the sensitivity to and therapeutic efficacy of cisplatin-based bladder cancer chemotherapy.

SFN has been shown to reverse the resistance of ovarian carcinoma cells to cisplatin by inducing DNA damage and the accumulation of intracellular cisplatin [14]. SFN also decreased drug resistance to cisplatin in cholangiocarcinoma cells [15] and in an in vivo lung cancer model [16]. Furthermore, SFN synergistically augmented the gemcitabine-mediated attenuation of viability and proliferation of cholangiocarcinoma cells [17]. Thus, beneficial characteristics of SFN with both gemcitabine and cisplatin have been demonstrated. The actual resistance problem, however, lies with cisplatin rather than gemcitabine as gemcitabine monotherapy has become obsolete. Since cisplatin serves as a first-line drug in treating numerous malignancies, it would be worthwhile to examine SFN’s effects on other tumors with established cisplatin resistance. SFN has also been demonstrated to synergistically enhance apoptosis and suppress the proliferation of lung cancer cells treated with carboplatin [18]. Since carboplatin often replaces cisplatin to mitigate severe side effects or accommodate for comorbidity including renal dysfunction, ongoing studies should also include the effects of SFN on tumor cells with carboplatin resistance.

Although chemotaxis was suppressed by SFN in all bladder cancer cell lines, adhesion to immobilized collagen and fibronectin was not uniformly suppressed. Instead, different responses were observed, depending on the cell line and whether the cell line was drug resistant or not. A similar phenomenon has recently been observed where SFN (2.5 µM) reduced the attachment of RT112 to collagen but elevated the attachment of TCCSUP cells [9]. These differences may possibly be traced back to integrin β1 and β4 expressions, which were differently modified by SFN in the different cell lines. The kind of integrin alteration also depended on the SFN incubation time (24 vs. 72 h exposure to SFN), pointing to dynamic integrin modifications. Such temporal sequences of integrin up- and down-regulation are not uncommon and are required to coordinate cell trafficking [19]. SFN altered integrin β1 and β4 such that the bladder cancer cell lines investigated here switched from collagen to fibronectin binding (e.g., partially RT112), lost contact to collagen and/or fibronectin (e.g., partially T24, TCCSUP), and/or firmly attached to matrix proteins (e.g., partially RT4). Thus, the number of transmigrated cells was reduced, either due to a loss of attached cells able to migrate or enhanced (firm) attachment that prevented motile spreading.

The relevance of β1 and β4 blockades to tumor cell adhesion was not investigated, but that to chemotaxis was. The β1 blockade resulted in reduced chemotaxis, except for RT112^par^, RT112^cis^, T24^gem^, and TCCSUP^gem^, whereas the β4 blockade triggered both the up- or down-regulation of chemotaxis, depending on the cell type and kind of resistance. Such inconsistent effects have already been documented in tumor cell adhesion and integrin profile analyses. Since adhesion proceeds after chemotactic tumor cell invasion, it is not surprising that the inhomogeneous integrin alterations caused by SFN are reflected in the integrin-blocking study. Nevertheless, increased chemotaxis of RT112^par^ and TCCSUP^gem^ following β4 blockade requires critical attention. β4 expression increased with RT112^par^ after 72 h of SFN incubation. This response might explain why the reduction in β4 on RT112^par^ was associated with increased chemotaxis of this cell line. Another scenario became apparent with TCCSUP^gem^, where β4 expression was only slightly lowered after 24 h of SFN exposure and not altered after 72 h. Enhanced chemotaxis of TCCSUP^gem^ following β4 blockade could, therefore, be an undesired counter effect or just an unspecific epiphenomenon and requires further investigation.

E-cadherin, N-cadherin, and vimentin are also involved in tumor cell invasion processes. E-cadherin was detected in RT112 and RT4, but not in T24 and TCCSUP cells. N-cadherin was absent in RT4 cells. T24 and TCCSUP cells had no E-cadherin but did have N-cadherin. RT4 and RT112 are well-differentiated (RT4) or moderately differentiated (RT112) urothelial cell carcinomas, whereas T24 and TCCSUP are derived from a poorly differentiated bladder carcinoma (grade 3/grade 4). Hence, E-/N-cadherin expression levels seem to reflect the aggressive phenotype of the tumor cell with E-cadherin pointing to an epithelial and N-cadherin to a mesenchymal phenotype. SFN up-regulated E-cadherin in RT112 cells (all sublines) and down-regulated N-cadherin in RT112 (all sublines), T24 and TCCSUP (both parental and cisplatin-resistant). The SFN-induced modulation of protein expression related to epithelial–mesenchymal translocation may explain its inhibitory action on chemotactic movement. Still, N-cadherin was not reduced by SFN in T24^gem^ and TCCSUP^gem^, even though chemotaxis was diminished. This indicates that something besides N-cadherin expression is responsible for regulating bladder cancer cell invasion.

Since a diminished E-cadherin expression appears to be associated with a more invasive phenotype, the decrease in E-cadherin observed in RT4 cells after SFN exposure was unexpected. The loss of E-cadherin cannot be correlated to the invasive capacity of RT4 since RT4 cells did not migrate in the chemotaxis assay. We postulate that E- and N-cadherin modulation by SFN depends on the initial protein composition of the tumor cells, whereby well-differentiated RT4 cells (strongly E-cadherin positive, N-cadherin negative) stand in opposition to poorly differentiated tumor cells, particularly TCCSUP and T24 (E-cadherin negative, N-cadherin positive). Other investigations have shown that the induction of growth and mesenchymal–morphological changes in RT4 cells was not accompanied by E-cadherin down-regulation [20], and driving RT4 cells to become invasive was even associated with an increase in E-cadherin protein in the early stimulation period [21]. Therefore, E-cadherin may also play a tumor-promoting role and drive resistance to chemotherapy [22]. This might explain why E-cadherin expression was highest in the cisplatin- and gemcitabine-resistant RT4 cells. Although SFN blocked the chemotaxis of all bladder cancer cell lines equally well, ongoing studies should further evaluate the molecular mechanisms of SFN in the early and late stages of the disease.

Cadherin localization differed in the different cell lines. The E-cadherin of RT112^par^ cells untreated with SFN was located in the plasma membrane. However, the E-cadherin of untreated RT112^cis^ and RT112^gem^ cells was enriched in the cytoplasm. Cytoplasmic E-cadherin expression has been shown to be a predictor of chemoresistance [23], which may explain the antipodal distribution of this protein in drug-sensitive and drug-resistant RT112 cells. E-cadherin was translocated from the cell membrane into the cell cytoplasm following the SFN treatment of RT112^par^ cells, whereas the opposite occurred following the SFN treatment of RT112^cis^ and RT112^gem^ cells. Since the chemotaxis of all RT112 sublines was reduced equally well by SFN, E-cadherin might be involved in chemotaxis curtailment in a dualistic manner. However, the initial E-cadherin localization (membranous or cytoplasmic) may determine its localization after exposure to SFN. The growth blockade of anaplastic thyroid cancer cells with initially high cytoplasmic E-cadherin accumulation was accompanied by a translocation of E-cadherin to the cell membrane [24]. Inversely, the application of the HDAC-inhibitor butyrate to oral squamous cell carcinoma cells with initially high membrane-bound E-cadherin promoted E-cadherin translocation to the cytoplasm [25]. Artesunate, an antimalarial, has been shown to block tumor growth in the colorectal cancer cell lines Lovo (poorly differentiated) and HT-29 (well differentiated). The growth inhibition was accompanied by appositional E-cadherin translocation, which refers to the shifting of E-cadherin to the membrane (Lovo) or into the cytoplasm (HT-29) [26]. This indicates that the distribution and trafficking of E-cadherin depends on the tumor cell differentiation status. Concurrently, we have found that SFN induces the translocation of E-cadherin from the membrane into the cytosol in the moderately differentiated RT112^par^ cells, but induces the translocation of E-cadherin from the cytosol to the membrane in the chemo-resistant, more aggressive RT112^cis^ and RT112^gem^ cell types.

The role N-cadherin plays in RT112 cells is ambivalent, since no striking difference of N-cadherin distribution was observed between cells not exposed to SFN and cells exposed to SFN. RT112 is a well-differentiated tumor with low N-cadherin and high E-cadherin expression levels. As other investigators have already reported, N-cadherin might be of minor importance in well-differentiated bladder cancer cells [27]. In contrast, N-cadherin translocation in poorly differentiated T24 cells (high N-cadherin expression) became evident under SFN exposure. Here, N-cadherin was lost at the cell membrane and enriched in the cytoplasm of T24^cis^ and T24^gem^ cells. The N-cadherin surface expression in chemo-resistant bladder cancer might play a role in tumor progression and the decrease in surface N-cadherin through SFN; this might be one mechanism contributing to the reduced migration capacity of (at least) the drug-resistant T24 cells. The translocation of N-cadherin in T24 cells has recently been demonstrated to be associated with reduced migration [27]. This was not apparent in drug-sensitive T24 cells, since N-cadherin of the untreated cells was already accumulated in the cytoplasm. Presumably, N-cadherin internalization under SFN depends on the initial N-cadherin expression level, which is highest in the drug-resistant cells.

Vimentin expression requires consideration since we found up-regulation when T24 cells were treated with SFN. This contrasts with reports indicating a correlation between vimentin expression and acquiring an invasive and metastatic bladder cancer phenotype [28]. Other investigators have demonstrated that spatial vimentin organization is critical for mediating cell migration [29]. Based on an investigation by Li and coworkers, vimentin accumulation at the leading edge of the tumor cells might be a prerequisite for beginning dynamic invasion [30]. Our findings are in line with this postulate. Vimentin was shown to be enriched at the edge of the T24 cell membrane but uniformly distributed on the cell surface following SFN application. Whether this reallocation contributes to a loss of motility or even to complete dysfunction, as assumed by others [31], remains open. Kuburich et al. have argued that the co-expression of vimentin and cytokeratin might be critical to carcinoma progression [32]. We found that SFN increased vimentin in T24^par^ and T24^gem^ cells, and this was paralleled by a loss of cytokeratin 8/18.

The findings presented here are based on in vitro models. In vivo research and clinical trials are needed to prove whether the integration of SFN into clinical treatment may actually delay or overcome cisplatin resistance. To optimize the bioavailability of SFN, conjugation to monoclonal antibodies or targeted drugs might be an innovative step towards implementing its clinical use.

## 5. Conclusions

SFN blocks adhesion and the chemotaxis of chemo-sensitive and cisplatin- and gemcitabine-resistant bladder cancer cell lines in vitro. The integrins β1 and β4 are involved in this blocking process, although their precise mode of action requires further evaluation. The cytoskeletal proteins E- and N-cadherin and vimentin are altered by SFN, with modulation of total protein expression and translocation between the cell membrane and cytoplasm. Therefore, SFN demonstrates potential for enhancing therapeutic efficacy in both chemo-sensitive bladder cancer and when cisplatin or gemcitabine resistance has developed.

## Data Availability

Data are contained within the article.

## Supplementary Materials

The following supporting information can be downloaded at: https://www.mdpi.com/article/10.3390/nu16050623/s1, Figure S1: Western blots.

## References

[1] Ferlay J., Colombet M., Soerjomataram I., Parkin D.M., Piñeros M., Znaor A., Bray F. (2021). Cancer statistics for the year 2020: An overview. Int. J. Cancer.

[2] Wang M., Zhang Z., Li Z., Zhu Y., Xu C. (2023). E3 ubiquitin ligases and deubiquitinases in bladder cancer tumorigenesis and implications for immunotherapies. Front. Immunol..

[3] Bonucci M., Geraci A., Pero D., Villivà C., Cordella D., Condello M., Meschini S., Del Campo L., Tomassi F., Porcu A. (2022). Complementary and Integrative Approaches to Cancer: A Pilot Survey of Attitudes and Habits among Cancer Patients in Italy. Evid. Based Complement. Altern. Med..

[4] Kanimozhi T., Hindu K., Maheshvari Y., Khushnidha Y.G., Kumaravel M., Srinivas K.S., Manickavasagam M., Mangathayaru K. (2021). Herbal supplement usage among cancer patients: A questionnaire-based survey. J. Cancer Res. Ther..

[5] Kennelley G.E., Amaye-Obu T., Foster B.A., Tang L., Paragh G., Huss W.J. (2023). Mechanistic review of sulforaphane as a chemoprotective agent in bladder cancer. Am. J. Clin. Exp. Urol..

[6] Schepici G., Bramanti P., Mazzon E. (2020). Efficacy of Sulforaphane in Neurodegenerative Diseases. Int. J. Mol. Sci..

[7] Xie H., Rutz J., Maxeiner S., Grein T., Thomas A., Juengel E., Chun F.K., Cinatl J., Haferkamp A., Tsaur I. (2022). Plant-Derived Sulforaphane Suppresses Growth and Proliferation of Drug-Sensitive and Drug-Resistant Bladder Cancer Cell Lines In Vitro. Cancers.

[8] Wang F., Liu P., An H., Zhang Y. (2020). Sulforaphane suppresses the viability and metastasis, and promotes the apoptosis of bladder cancer cells by inhibiting the expression of FAT-1. Int. J. Mol. Med..

[9] Justin S., Rutz J., Maxeiner S., Chun F.K., Juengel E., Blaheta R.A. (2020). Bladder Cancer Metastasis Induced by Chronic Everolimus Application Can Be Counteracted by Sulforaphane In Vitro. Int. J. Mol. Sci..

[10] He C., Buongiorno L.P., Wang W., Tang J.C.Y., Miceli N., Taviano M.F., Shan Y., Bao Y. (2021). The Inhibitory Effect of Sulforaphane on Bladder Cancer Cell Depends on GSH Depletion-Induced by Nrf2 Translocation. Molecules.

[11] Mokhtari R.B., Qorri B., Baluch N., Sparaneo A., Fabrizio F.P., Muscarella L.A., Tyker A., Kumar S., Cheng H.M., Szewczuk M.R. (2021). Next-generation multimodality of nutrigenomic cancer therapy: Sulforaphane in combination with acetazolamide actively target bronchial carcinoid cancer in disabling the PI3K/Akt/mTOR survival pathway and inducing apoptosis. Oncotarget.

[12] Liu F., Lv R.B., Liu Y., Hao Q., Liu S.J., Zheng Y.Y., Li C., Zhu C., Wang M. (2020). Salinomycin and Sulforaphane Exerted Synergistic Antiproliferative and Proapoptotic Effects on Colorectal Cancer Cells by Inhibiting the PI3K/Akt Signaling Pathway in vitro and in vivo. OncoTargets Ther..

[13] Bagheri M., Fazli M., Saeednia S., Gholami Kharanagh M., Ahmadiankia N. (2020). Sulforaphane Modulates Cell Migration and Expression of β-Catenin and Epithelial Mesenchymal Transition Markers in Breast Cancer Cells. Iran. J. Public Health.

[14] Gong T.T., Liu X.D., Zhan Z.P., Wu Q.J. (2020). Sulforaphane enhances the cisplatin sensitivity through regulating DNA repair and accumulation of intracellular cisplatin in ovarian cancer cells. Exp. Cell Res..

[15] Račkauskas R., Zhou D., Ūselis S., Strupas K., Herr I., Schemmer P. (2017). Sulforaphane sensitizes human cholangiocarcinoma to cisplatin via the downregulation of anti-apoptotic proteins. Oncol. Rep..

[16] Li Q.Q., Xie Y.K., Wu Y., Li L.L., Liu Y., Miao X.B., Liu Q.Z., Yao K.T., Xiao G.H. (2017). Sulforaphane inhibits cancer stem-like cell properties and cisplatin resistance through miR-214-mediated downregulation of c-MYC in non-small cell lung cancer. Oncotarget.

[17] Tomooka F., Kaji K., Nishimura N., Kubo T., Iwai S., Shibamoto A., Suzuki J., Kitagawa K., Namisaki T., Akahane T. (2023). Sulforaphane Potentiates Gemcitabine-Mediated Anti-Cancer Effects against Intrahepatic Cholangiocarcinoma by Inhibiting HDAC Activity. Cells.

[18] Chatterjee S., Rhee Y.H., Ahn J.C. (2016). Sulforaphene-Carboplatin Combination Synergistically Enhances Apoptosis by Disruption of Mitochondrial Membrane Potential and Cell Cycle Arrest in Human Non-Small Cell Lung Carcinoma. J. Med. Food.

[19] Siech C., Rutz J., Maxeiner S., Grein T., Sonnenburg M., Tsaur I., Chun F.K., Blaheta R.A. (2022). Insulin-like Growth Factor-1 Influences Prostate Cancer Cell Growth and Invasion through an Integrin α3, α5, αV, and β1 Dependent Mechanism. Cancers.

[20] Li Y., Yang X., Su L.J., Flaig T.W. (2010). VEGFR and EGFR inhibition increases epithelial cellular characteristics and chemotherapy sensitivity in mesenchymal bladder cancer cells. Oncol. Rep..

[21] Mehus A.A., Bergum N., Knutson P., Shrestha S., Kalonick M., Zhou X., Garrett S.H., Sens D.A., Sens M.A., Somji S. (2022). Chronic Arsenic Exposure Upregulates the Expression of Basal Transcriptional Factors and Increases Invasiveness of the Non-Muscle Invasive Papillary Bladder Cancer Line RT4. Int. J. Mol. Sci..

[22] Rubtsova S.N., Zhitnyak I.Y., Gloushankova N.A. (2022). Dual role of E-cadherin in cancer cells. Tissue Barriers.

[23] Bendardaf R., Sharif-Askari F.S., Sharif-Askari N.S., Syrjänen K., Pyrhönen S. (2019). Cytoplasmic E-Cadherin Expression Is Associated With Higher Tumour Level of VEGFA, Lower Response Rate to Irinotecan-based Treatment and Poorer Prognosis in Patients With Metastatic Colorectal Cancer. Anticancer Res..

[24] Xiong L., Nie J.H., Lin X.M., Wu J.B., Chen Z., Xu B., Liu J. (2020). Biological implications of PTEN upregulation and altered sodium/iodide symporter intracellular distribution in resveratrol-suppressed anaplastic thyroid cancer cells. J. Cancer.

[25] Zang W., Liu J., Geng F., Liu D., Zhang S., Li Y., Pan Y. (2022). Butyrate promotes oral squamous cell carcinoma cells migration, invasion and epithelial-mesenchymal transition. PeerJ.

[26] Li L.N., Zhang H.D., Yuan S.J., Yang D.X., Wang L., Sun Z.X. (2008). Differential sensitivity of colorectal cancer cell lines to artesunate is associated with expression of beta-catenin and E-cadherin. Eur. J. Pharmacol..

[27] Wint H., Li J., Abe T., Yamada H., Higaki T., Nasu Y., Watanabe M., Takei K., Takeda T. (2023). Pacsin 2-dependent N-cadherin internalization regulates the migration behaviour of malignant cancer cells. J. Cell Sci..

[28] Monteiro-Reis S., Miranda-Gonçalves V., Guimarães-Teixeira C., Martins-Lima C., Lobo J., Montezuma D., Dias P.C., Neyret-Kahn H., Bernard-Pierrot I., Henrique R. (2023). Vimentin epigenetic deregulation in Bladder Cancer associates with acquisition of invasive and metastatic phenotype through epithelial-to-mesenchymal transition. Int. J. Biol. Sci..

[29] Wang Y., Gong J., Yao Y. (2020). Extracellular nanofiber-orchestrated cytoskeletal reorganization and mediated directional migration of cancer cells. Nanoscale.

[30] Li S., Xiong N., Peng Y., Tang K., Bai H., Lv X., Jiang Y., Qin X., Yang H., Wu C. (2018). Acidic pHe regulates cytoskeletal dynamics through conformational integrin β1 activation and promotes membrane protrusion. Biochim. Biophys. Acta Mol. Basis Dis..

[31] Frescas D., Roux C.M., Aygun-Sunar S., Gleiberman A.S., Krasnov P., Kurnasov O.V., Strom E., Virtuoso L.P., Wrobel M., Osterman A.L. (2017). Senescent cells expose and secrete an oxidized form of membrane-bound vimentin as revealed by a natural polyreactive antibody. Proc. Natl. Acad. Sci. USA.

[32] Kuburich N.A., den Hollander P., Pietz J.T., Mani S.A. (2022). Vimentin and cytokeratin: Good alone, bad together. Semin. Cancer Biol..

