# Supplementary figures and images for: Sulforaphane Inhibits Adhesion and Migration of Cisplatin- and Gemcitabine-Resistant Bladder Cancer Cells In Vitro
Source: Nutrients. 2024 Feb 23;16(5):623. doi: 10.3390/nu16050623 (PMC10934724; doi:10.3390/nu16050623)

Figure S1. Western blots.

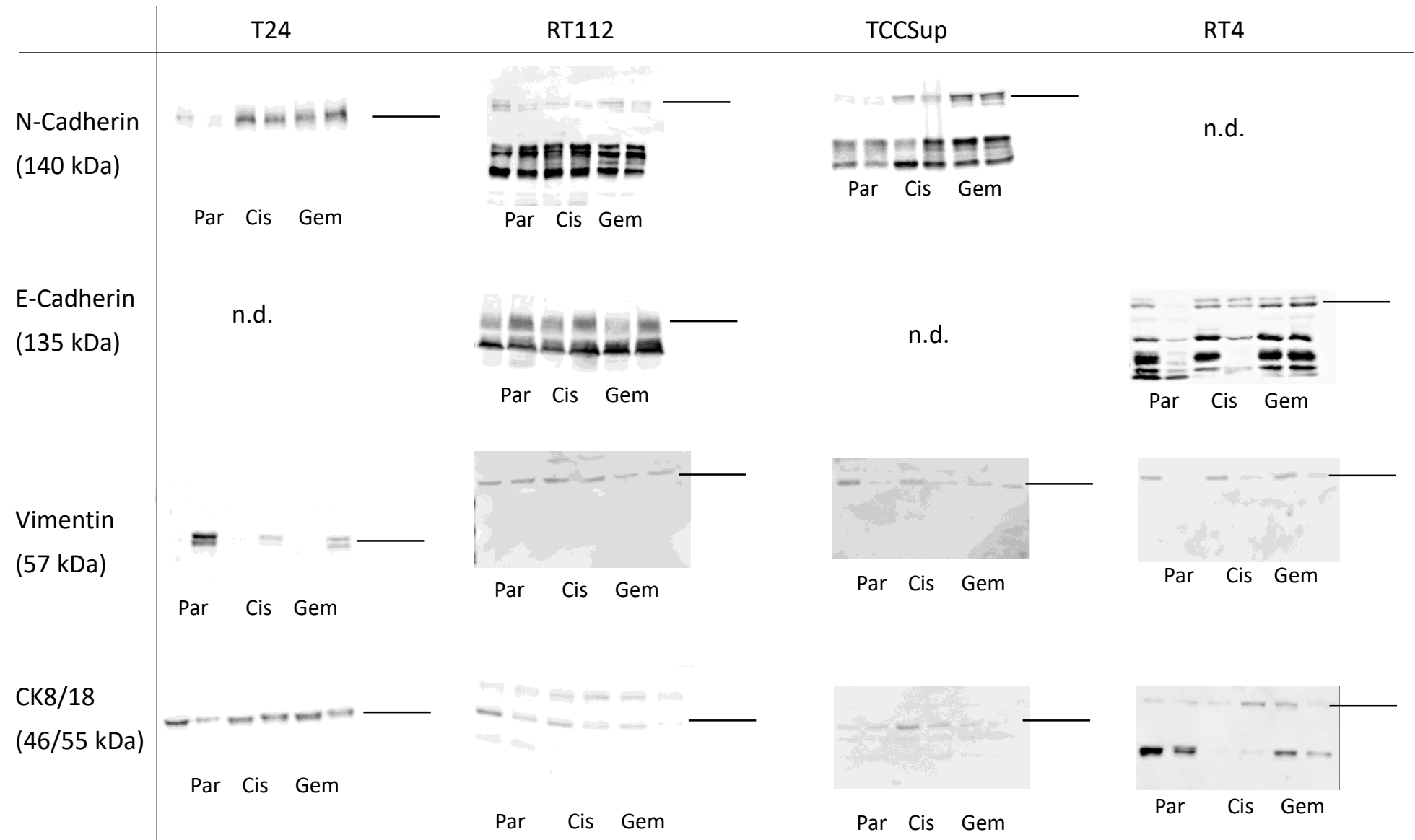

Supplement: Supplementary file 1 [file nutrients-16-00623-s001.zip › nutrients-2851223-supplementary.pdf]
